# Taxonomy of the *Cryptopygus* complex. I. *Pauropygus* - a new worldwide littoral genus (Collembola, Isotomidae)

**DOI:** 10.3897/zookeys.304.4083

**Published:** 2013-05-22

**Authors:** Mikhail Potapov, Yan Gao, Louis Deharveng

**Affiliations:** 1 Moscow State Pedagogical University, Kibalchich str., 6, korp. 5, Moscow, 129164 Russia; 2 Institute of Plant Physiology & Ecology, Shanghai Institutes for Biological Sciences, Chinese Academy of Sciences, Shanghai 200032, China; 3 UMR7205 CNRS, Origine, Structure et Evolution de la Biodiversité, Museum National d’Histoire Naturelle, 75005-Paris, France

**Keywords:** New genus, modified mouthparts, sensillar chaetotaxy, littoral habitat

## Abstract

In this paper, we describe the new genus *Pauropygus* **gen. n.** which includes three minute species, blind and unpigmented, living in interstitial littoral habitats in tropical or subtropical countries. Two of these species are new to science (type species *Pauropygus projectus* **sp. n.** from New Caledonia and *Pauropygus pacificus* **sp. n.** from China); the third one, originally described in the genus *Cryptopygus* (*Cryptopygus caussaneli* Thibaud, 1996), has a larger pantropical distribution. We synonymize here *Cryptopygus riebi* Barra, 1997 from South Africa with *Pauropygus caussaneli*. Two paratypes of the Mexican species *Cryptopygus axayacatl* Palacios & Thibaud, 2001 turned also to be *Pauropygus caussaneli*, while the holotype and remaining paratypes of this species support its placement in *Proisotomodes*. Among the *Cryptopygus* complex, *Pauropygus* gen. n. is easily recognized by characters of mouthparts (presence of two large projections on pleural fold, basolateral field with 6 chaetae, modified mouthparts) and reduced sensillar chaetotaxy (tergal sensilla 2-3,0-1/0-1,0-1,1-2,1-2,1-3, microsensilla reduced in number: 00/0-100, with sensilla situated in p-row on the abdomen). Small size, absence of eyes and pigment are also shared by all its species. The three species belonging to the genus differ by sensillar chaetotaxy.

## Introduction

The species of the subfamily Anurophorinae with furca and two last abdominal segments fused are widely distributed in the world. If special remarkable detail had not been found in such species, they were usually assigned to genera *Isotomina* Börner, 1903 (mostly in Northern Hemisphere) or *Cryptopygus* Willem, 1902 (mostly in Southern Hemisphere). In the course of the development of taxonomy in the family Isotomidae the question of division of these two artificial taxa became important. So far *Isotomina*, *Cryptopygus* and several formally similar genera have been given some discussions and historical reviews (Deharveng 1981; Potapov 2001; Rusek 2002; Deharveng et al. 2005; Potapov et al. 2009, Jordana et al. 2009). Most forms, however, of this so-called “*Cryptopygus* complex” did not find their final generic position and therefore even modern monographs and catalogues continue to use *Cryptopygus* in a wide sense (Fjellberg 2007; Danyi and Traser 2008; Furuno et al. 2000, and others). At convenience, we accept the traditional understanding of the complex - it means Abd.V and VI fused (vs. separated in *Proisotoma* complex). We believe that fusion/separation of Abd.V and VI is really a convenient feature to separate these two large groups of taxa of which the latter one is more common in Northern Hemisphere while the former mostly occupies Southern Hemisphere. In the present paper, we describe a new genus for three species, two of which are new to science and share several remarkable morphological modifications that are probably adaptations to interstitial seashore habitats.

### Abbreviations used in the descriptions

AIIIO apical organ of the third antennal segment, Abd. abdominal segment, Ant. antennal segment, Th. thoracic segment, Ti. tibiotarsus, U. unguis, VT ventral tube

## Taxonomy

### Pauropygus gen. n.

urn:lsid:zoobank.org:act:FD85013E-80AF-46F9-9BC9-564339BF37E5

http://species-id.net/wiki/Pauropygus

#### Type species:

*Pauropygus projectus* sp. n.

#### Diagnosis.

Blind small-sized Anurophorinae with two last abdominal segment fused, modified mouthparts including remarkably modified pleural fold, and first segments of antenna set together on frontal part of head.

#### Description.

**Figures 1–7.**
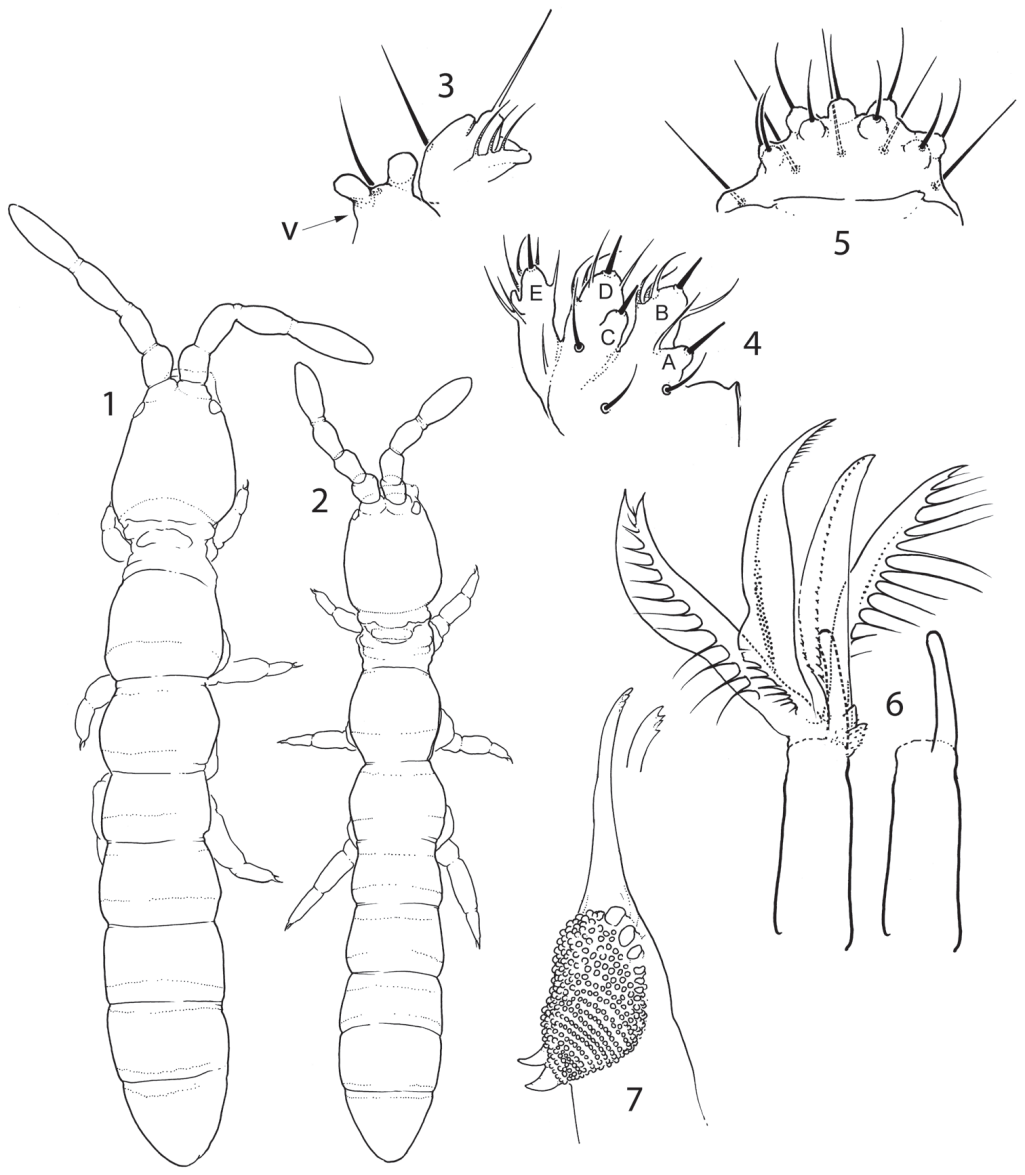
*Pauropygus projectus* sp. n. (**1, 3–7**) and *Pauropygus caussaneli* (**2**). **1–2** general habitus (dorsally) **3** maxillary outer lobe and pleural fold (v: v-shaped process.) **4** labial palp **5** labrum **6** maxillary head (on right, basal part and claw shown) **7** mandible.

**Figures 8–12.**
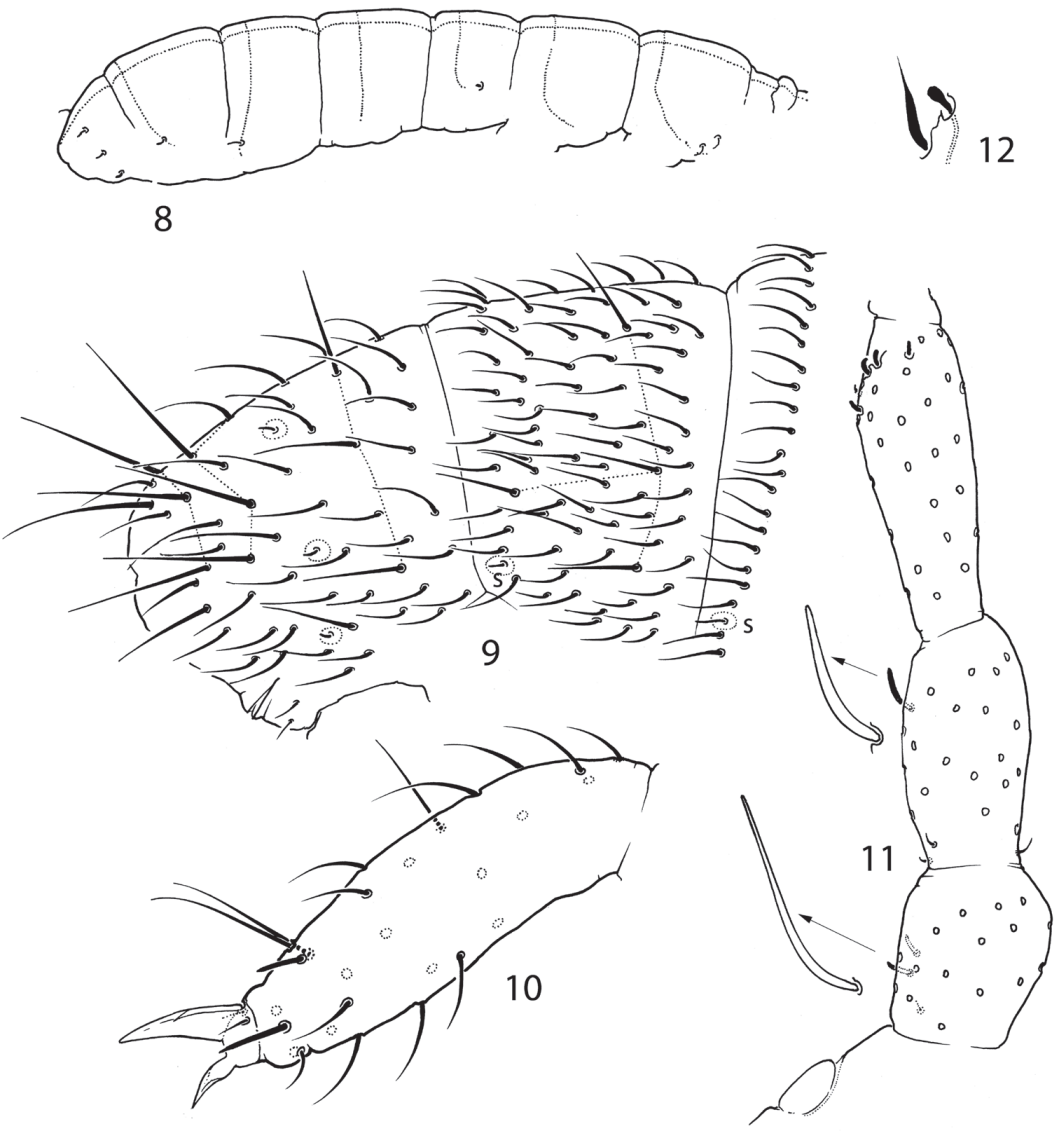
*Pauropygus projectus* sp. n. **8** sensillar chaetotaxy **9** posterior part of abdomen (s – sensillum) **10** distal half of Leg III **11** PAO and Ant.I-III, dorsal view **12** subapical organite and microchaeta.

Without pigment and eyes, Abd.V and VI fused. Body size small, with primary granulation only. Antennal bases set close together on frontal side of the head, almost touching each other (Figs 1, 2). Sensilla on three first antennal segments thickened. Sensilla of Ant.IV moderately thickened. Maxillary palp simple, with 3 sublobal hairs set together (Fig. 3). Pleural fold with two high projections (Figs 3, 20, 23). Basolateral field of the labium with 6 chaetae (Fig. 20). Labium with three papilla projected forward, number of guards not reduced. Papillate sensilla reduced in size. Labrum swollen in central part, labral chaetae set on wide papilla. Two prelabral chaetae. Maxillary head with four enlarged lamellae, three of them ciliated; claw reduced to small finger-like process, not dentate. Mandible head reduced and thin, molar plate with 2 strong basal teeth. Tergal sensilla on abdomen situated in p-row of chaetae, their number reduced (2-3,0-1/0-1,0-1,1-2,1-2,1-3, depending on species), number of microsensilla 00/100 or 00/000 (Figs 8, 15, 21). Th.I-III without ventral chaetae. Body macrochaetae differentiated. Tibiotarsal tenent chaetae present (1-2-2), not clavate. VT with 4+4 laterodistal chaetae. Tenaculum with 4+4 teeth. Furca slender, manubrium with a pair of chaetae on anterior side, dens with crenulation and wide swelling on posterior side, mucro bidentate.

#### Name derivation.

The name is derived from the Greek suffix –pygus which points to the fusion of the abdominal segments and from the Greek prefix pauro- which refers to the reduced chaetotaxy, particularly reduced number of sensilla on the body.

#### Representatives.

Apart from the type species *Pauropygus projectus* sp. n., the new genusincludes *Cryptopygus caussaneli* Thibaud, 1996 and *Pauropygus pacificus* sp. n.

#### Affinity.

The new genus is established mainly due to the unusual position of antennae on head and the strongly modified mouthparts. In mouthparts, the V-shaped pleural folds and the presence of 6 chaetae on basolateral fields of the labium are especially important; these characters were never seen in the family before, except for the latter one that was mentioned for part of the Algerian population of *Isotominella geophila* sensu Jordana et al., 2009; normally, the pleural fold looks like a weak swelling proximal to the maxillary outer lobe, and basolateral field of mouth cone has 5 chaetae in species of the Isotomidae family (Fjellberg 1984, 1999). Other modifications of mouthparts, like swollen labrum, unequal labial papilla, reduced plate of outer lobe of maxilla, are more common features. The sixth chaeta of basolateral field has unclear derivation; since it has weaker socket than the other five, it is probably one of the sublobal hairs which has migrated from the sublobal plate to more posterior area of head and grouped together with basolateral chaetae. Together with two finger-like extensions of pleural folds this chaeta probably makes lateral parts of head more functionally important. The projected position of antennae and modified mouthparts are probably adaptations to an interstitial life between small sand grains on the beach and to feeding on particles suspended in water. Projections on different parts of body are well known among species living in contact with salt water in genera *Archisotoma* Linnaniemi, 1912, *Anuridella* Willem, 1906, *Xenylla* Tullberg, 1869, *Hypogastrura* Bourlet, 1839, *Friesea* Dalla Torre, 1895, and others.

As an unusual feature for the group, the species of the new genus show considerable reduction of sensillar chaetom. In *Pauropygus projectus* sp. n. all medial sensilla on Th.II-Abd.IV and lateral sensilla on Th.III-Abd.II are lost, while in *Pauropygus caussaneli* and *Pauropygus pacificus* sp. n. it is the posterior and lateral parts of body that lost sensilla. In spite of differences between number of sensilla on body (2,0/0,0,1,1,3) and (3,1/1,1,2,2,3) among *Pauropygus* species, the general pattern of their distribution and differentiation is kept.

*Pauropygus* is closely related to *Isotominella* Delamare Deboutteville, 1948 after the redescription of Jordana et al. (2009). The identity of the type specimens of *Isotominella geophila* Delamare Deboutteville, 1948 (Ivory Coast) which were not seen by Jordana and specimens from Algeria on which the redefinition was based remains somewhat doubtful. The two genera share simple maxillary palp, two prelabral chaetae, posterior position of sensilla on tergites, and general appearance of furca. Other shared characters (blindness, absence of foil chaetae, sensillar equipment of antennae, microsensillar set 10/100) are less significant. Apart from the two characters mentioned above, *Pauropygus* sp. n. differs from *Isotominella* in more differentiated tibiotarsal chaetotaxy (presence of tenent and spiny chaetae), shape of PAO (flat and broad vs. oval), number of sublobal hairs and e-guards (3 vs. 2, and 7 vs. 5, respectively). *Isotominella* also has a rather common set of sensilla on body (33/22223) while it is reduced in *Pauropygus*. At last, the new genus is strictly restricted to seashore sands, while *Isotominella geophila* prefers soil. We also examined specimens of *Isotominella geophila* from Algeria kindly provided for us by Jordana and surprisingly concluded that females and males also differ in antennae on head which are positioned almost like in *Pauropygus* in males and set apart in females. The crenulation of basal part of dens was stressed by both Delamare Debouteville (1948) and Jordana et al. (2009) as one, if not the main diagnostic characters of the genus *Isotominella*. We consider that this character is of low taxonomical value since it strongly depends on mounting of the animal on slide. We have also seen other specimens of *Isotominella* from Eurasia (Ukraine and China). They did not show the dimorphism of Algerian populations and represent at least one more species of the genus. The comparison of *Pauropygus* gen. n. with other genera of the *Cryptopygus* complex is given below.

### Pauropygus projectus sp. n.

urn:lsid:zoobank.org:act:4747ED35-9352-4F40-B567-598C47E6BF71

http://species-id.net/wiki/Pauropygus_projectus

Figs 1, 3 –14

#### Material.

Holotype female on slide and 13 paratypes (6 on slides, 7 in alcohol): New Caledonia: Iles Loyautés: Ouvéa island: Gossanah: plage de Hoony, collected by flotation from beach sand, 13.11.2000 (sample # NC00-252), leg L. Deharveng & A. Bedos. Coordinates: 166.632°E, 20.4365°S. Material is deposited in Museum National d’Histoire Naturelle, Paris (holotype and 9 paratypes, no male) andin Moscow State Pedagogical University (4 paratypes, including one adult male).

Other material. New Caledonia: Iles Loyautés: Tiga island: Toka village, collected by flotation from beach sand, 31.10.2000 (sample # NC00-145: two on slides; sample # NC00-146: one on slide), leg L. Deharveng & A. Bedos. Coordinates: 167.795704°E, 21.098038°S.

#### Description.

Size 0.5–0.6 mm. White, without eyes. Cuticle with thin hardly visible primary hexagonal granulation (“smooth”). PAO flat, roundish, not constricted, about 1.5 as long as inner edge of U.III and shorter than width of Ant.I (Fig. 11). Sublobal plate of maxillary outer lobe small with 3 hairs grouped together, palp simple. Pleural fold with one chaeta (as common for the family) and two high projections (Fig. 3). Labral chaetotaxy as 2/554, middle part of labrum swollen, chaetae in two apical rows set on wide papillae, edge of labrum weakly developed (Fig. 5). Labium with 3 proximal, 6 basolateral and 4 basomedian chaetae and a complete set of papillae (A-E) and guards (16). Papillae B, D and E projected considerably forward, papillae A and C partly reduced and fused with B and D, respectively (Fig. 4). Ventral side of head with 6-7+6-7 postlabial chaetae. Ventrolateral chaetae of head and postlabial chaetae delimit an almost unbroken unsetaceous area. Maxillary head elongated, with four well visible enlarged lamellae of which two have long cilia and two have fine serration. Two remaining lamellae possibly as small weakly serrated projections set in a common cluster at base of claw (Fig. 6). Maxillary claw reduced, single-tipped, finger-shaped, with some weak teeth at the head which are visible in lateral view only (not shown on figure). *Pars incisiva* of mandible slender, apically with four weak teeth, basal part of *pars molaris* with two strong hooks (Fig. 7).

Ant.I with many chaetae (more than 20), 1 ventro-basal microchaeta (bms; dorsal bms not differentiated), and 2 thick ventral sensilla (s). Ant.II with 3 rather large bms and 1 thick laterodistal s. Ant.III without bms and with 5 distal s of which two inner as thick and short as outer ones. Male antennal “spurs” unknown. Sensilla on Ant.IV weakly differentiated, as common for the family, subapical organite pin-like and small, subapical microsensillum short and curved (Figs 11, 12).

Dorsal axial chaetom of Th.II–Abd.III as 12-13,7-9/5(4),5(4),5(4). Macrochaetae erect, smooth and rather long, more differentiated laterally on Th.II–III and on posterior half of abdomen, with the whole number as 1,1/3,3,3,4 (Th.II-Abd.IV). Medial macrochaetae on Abd.V about 0.4 as long as dens. Sensilla on tergites clearly differentiated, significantly shorter and slightly thinner than ordinary chaetae. Sensillar formula 20/00113 (s), 00/100 (ms) (Fig. 8). Lateral sensilla on Abd.III, IV in posterior position. Sensilla on Th.II and Abd.III much longer than on Abd.V, sensilla on Abd.IV of medium size (Fig. 9).

**Figures 13–14:**
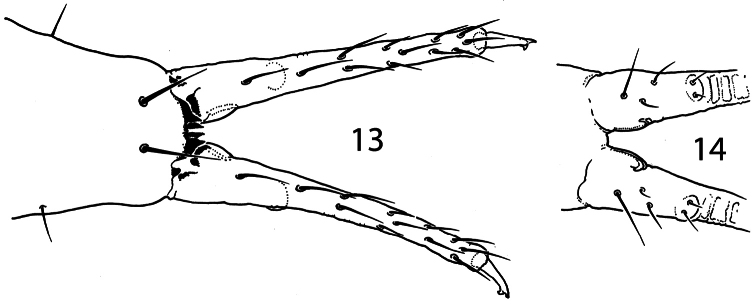
. *Pauropygus projectus* sp. n. anterior (**13**) and posterior (**14**, part) sides of furca.

Unguis of normal shape, without inner tooth, two lateral teeth forming a weak tunica partly covering dorsal edge of unguis (Fig. 10). Upper subcoxa of Leg I-III with 1,1,4 chaetae, lower subcoxa with 1,10,11 (one individual studied). Ti.I-III with basic set of chaetae (21-22, 22-23, 28), T-chaetae absent. Chaetae on tibiotarsi with irregular distribution, outer side with higher number of chaetae than inner side. Chaetae C7 (inner part of tibiotarsi) either lost or migrated laterally. Modification of chaetae x and B5 on Ti. III in males unknown (no males). Distal tibiotarsal tenent chaetae on Ti.I-III (1-2-2) well developed, not clavate, about 1.5 as long as U.III. Each tibiotarsus with one (sometimes two on Leg II) additional tenent chaeta at middle part. Ti.III with two stick-like chaetae (A6, A7) in distal ring. Ti.I,II with similar thickened chaetae but much less developed. One or two inner chaetae of distal ring of Ti.I-III shorter than others. Three chaetae of distal ring A (two tenent chaetae and one nearby) set apart from distal edge of tibiotarsus, unlike other chaetae of the ring (Fig. 10). Pretarsus with two chaetae, inner one shorter. Ventral tube with 4+4 laterodistal and 2 posterior chaetae. Tenaculum with 4+4 teeth and 1 chaeta. Anterior furcal subcoxa with 13–15 chaetae, posterior with 6-7. Furca of medium size. Anterior side of manubrium with a pair of distal chaetae, posterior one with 8+8 chaetae on the main part and 3+3 on the laterobasal lobes (one individual studied). Manubrial thickening with a pair of additional inner teeth. Lateral parts of manubrium with 1+1 chaetae. Dens slender, anteriorly with 12 (more rarely 11) chaetae. Posterior side of dens slightly crenulated in the medial part, with 5 chaetae of which 3 basal and 2 at the medial part (short inner and long outer) set together on wide papillum. Mucro slender with two teeth of which the subapical one is larger, lamellae not differentiated (Figs 13, 14). Ratio of manubrium: dens: mucro = 2.8–3.4: 4.5–4.6: 1. Anal lobes without microchaetae.

#### Affinity.

*Pauropygus projectus* sp. n. differs from *Pauropygus caussaneli* in number of sensilla on body (20/00113 vs. 31/11221, see also the genus affinity section), relative size of inner and outer sensilla in AIIIO (outer ones smaller than inner ones in *Pauropygus caussaneli*), number of posterior chaetae on ventral tube (2 vs. 4), serrated chaetae on upper and lower subcoxae (smooth in *Pauropygus caussaneli*), more differentiated chaetal equipment of legs. Besides, *Pauropygus projectus* sp. n. is larger in size, which may explain higher number of chaetae on Ant.I, parts of legs, axial group of tergites and postlabial area than in *Pauropygus caussaneli* (see descriptions for details).

#### Name derivation.

The species has well developed “projections” on the body as: swollen labrum, v-shaped pleural folds, extended labial palp, and antennae projected ahead.

#### Distribution.

Only from Loyalty Islands in New Caledonia.

### Pauropygus caussaneli

(Thibaud, 1996) comb. n.

http://species-id.net/wiki/Pauropygus_caussaneli

Figs 2 , 15 –20

Cryptopygus caussaneli Thibaud, 1996
Cryptopygus riebi Barra, 1997 n. syn.

#### Material.

Holotype and paratypes of *Cryptopygus caussaneli*: Mauritania (Aftout-es-Sahel); holotype and paratypes of *Cryptopygus riebi*: South Africa (Sodwana Bay, Natal Province); two paratypes of *Cryptopygus axayacatl* Palacios-Vargas & Thibaud, 2001: Mexico (Guerrero, Acapulco); material of J.-M.Thibaud identified by him as *Cryptopygus caussaneli* from Senegal, Morocco, Congo, Madagascar, Maurice and Mayotte Islands. More precise labels in the associated publications (Palacios-Vargas & Thibaud, 2001; Thibaud & Ndiaye, 2006; Thibaud & Boumezzough, 2006; Thibaud, 2008). All specimens are kept in the Museum national d’Histoire naturelle of Paris (France).

#### Redescription.

White, without eyes. Size up to 0.4 mm. Cuticle smooth. PAO about 1,9 as long as inner edge of U.III and shorter (0.7) than width of Ant.I. Outer (Fig. 20) and inner mouthparts principally as in *Pauropygus projectus*. Ventral side of head with 5+5 postlabial chaetae. Ant.I with 14-15 chaetae, 1 ventro-basal microchaeta (bms; dorsal bms not differentiated), and 2 thick ventral sensilla (s), short and long. Ant.II with 3 bms and 1 thick laterodistal s. Ant.III without bms and with 5 distal s of which two inner thicker and longer than outer ones. Male antennal “spurs” present on Ant.II, III and basal part of Ant.IV.

**Figures 15–18.**
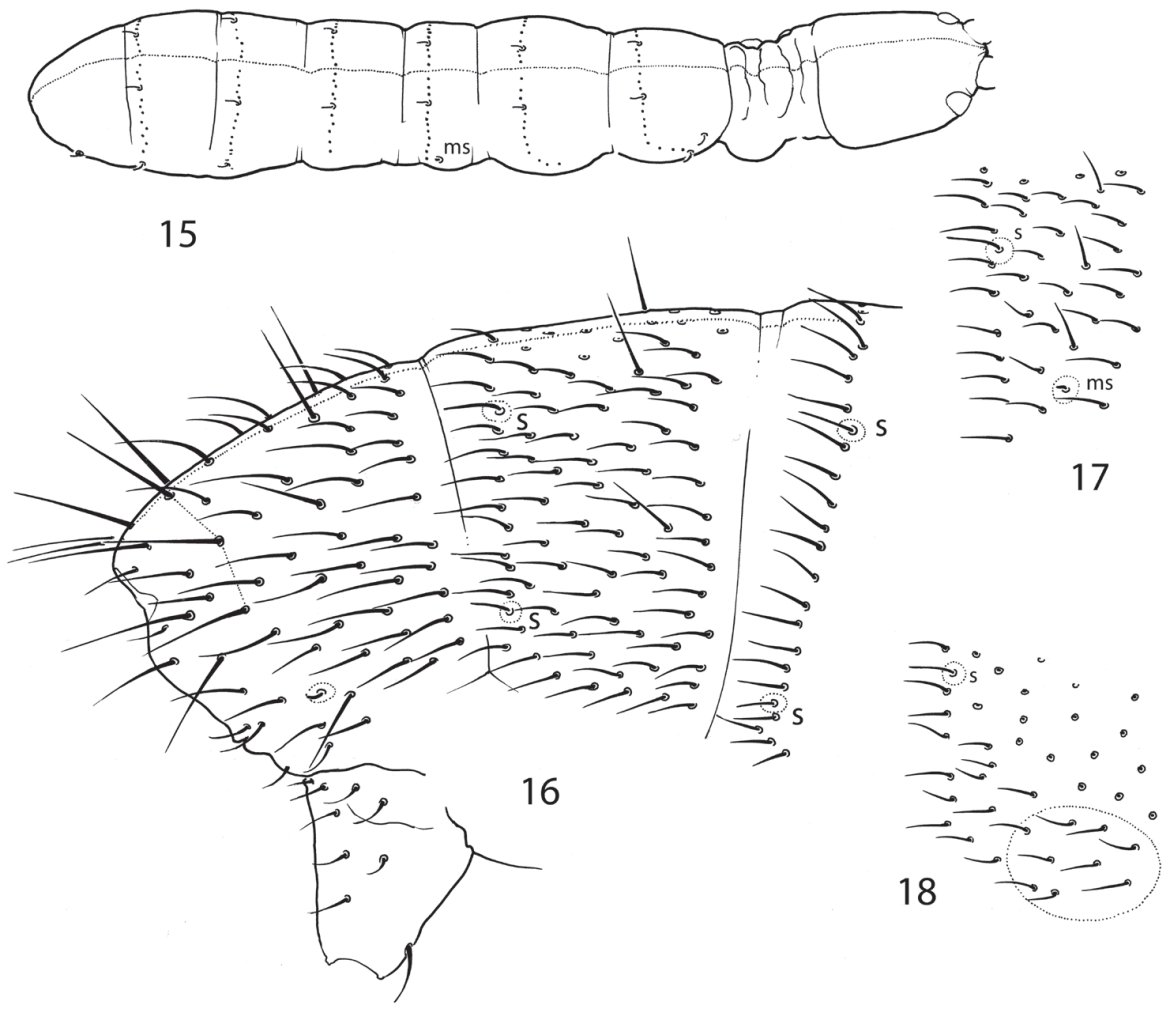
*Pauropygus caussaneli*. **15** sensillar chaetotaxy **16** posterior part of abdomen **17** lateral part of Abd.I **18** lateral part of Th.III. s sensillum, ms microsensillum.

Dorsal axial chaetom of Th.II–Abd.III as 10,6/4,4(3),4(3). Thorax without ventral chaetae. Macrochaetae smooth, with the whole number as 1(2),1(2)/3,3,3,4 (Th.II-Abd.IV). Medial macrochaetae on Th.II-Abd.III hardly differentiated. Medial macrochaetae on Abd.V about 0.4 as long as dens. All sensilla of thorax and medial sensilla of abdomen nearly as long as ordinary chaetae and hardly visible, lateral sensilla on Abd.III-V shorter than ordinary chaetae. Microsensilla short but well visible. Sensillar formula 31/11221 (s), 00/100 (ms) (Fig. 15-18). Sensilla in posterior position. (Fig. 16).

**Figures 19–20.**
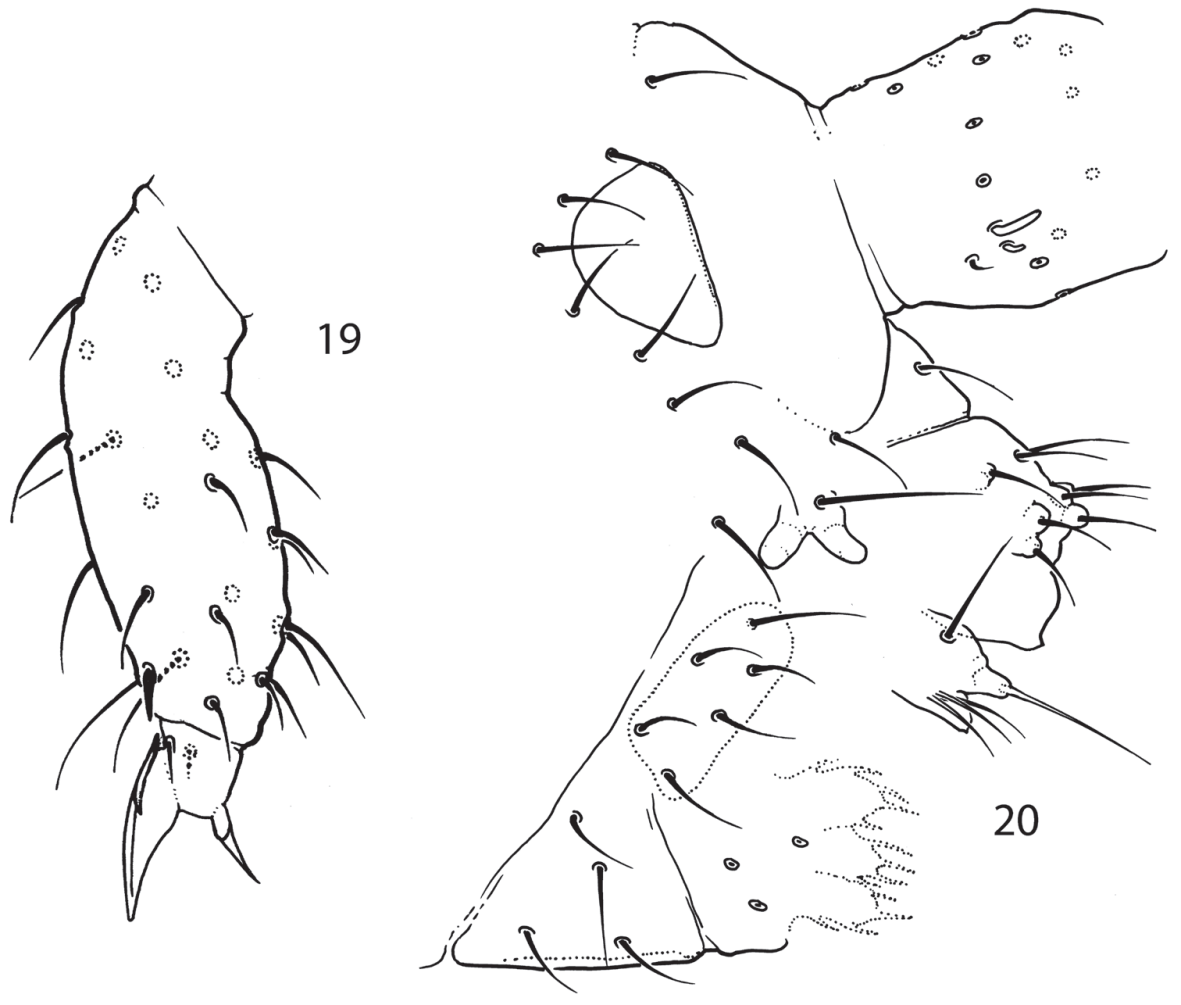
*Pauropygus caussaneli*. **19** distal half of Leg III **20** anterior part of head, lateral view (group of basolateral chaetae marked).

Unguis of normal shape, without inner tooth, two broad unequal lateral teeth. Upper subcoxa of Leg I-III with 1,1,4 chaetae, lower subcoxa with 1,6-7,7-8. Ti.I-III with one chaeta lost in basic set (20, 21, >25). Chaetae x and B5 on Ti. III in males unmodified. Distal tibiotarsal tenent chaetae on Ti.I-III (1-2-2) well developed, not clavate, about 1.1-1.2 as long as U.III. Each tibiotarsus with one additional tenent chaeta at middle part. Ti.III with one stick-like chaetae (A7) in distal ring, its shape slightly varies (Fig. 19). Tenent hairs (1,2,2) about 1.1–1.2 as long as U.III (Fig. 19). Pretarsus with two chaetae. Ventral tube with 4+4 laterodistal and 4 posterior chaetae in one transversal row. Tenaculum with 4+4 teeth and 1 chaeta. Anterior furcal subcoxa with 12–15 chaetae, posterior with 6 ones. Manubrium principally as in previous species. Dens slender, anteriorly normally with 11 chaetae. Posterior side of dens slightly crenulated in the medial part, with 5 chaetae of which 3 basal and 2 at the medial part (short inner and long outer) set together on low papillum. Mucro slender with two teeth of unequal size. Ratio of manubrium: dens: mucro = 3.2–3.6: 4.1–4.3: 1. Anal lobes without microchaetae.

#### Affinity.

See above for differences with *Pauropygus projectus* sp. n. After our study of type material of *Cryptopygus riebi* (South Africa) and two paratypes of *Cryptopygus axayacatl* (Mexico) we concluded that these species only complement the wide distribution area of *Pauropygus caussaneli*. Holotype and other paratypes of *Cryptopygus axayacatl* support its species status, but in the genus *Proisotomodes*.

#### Distribution.

Probably widely distributed on sandy seashores of tropics and subtro-pics. Recorded from the coast of Indian and Atlantic Oceans (Africa, Central America).

### Pauropygus pacificus sp. n.

urn:lsid:zoobank.org:act:AA6C41A0-CEAE-4961-B9EB-CC8AAB3B813C

http://species-id.net/wiki/Pauropygus_pacificus

Figs 21–24

#### Material.

Holotype: China, Shandong Province, Yantey City, sea coast near Tuchengzi Hills, sandy beach, collected by flotation of thin sand under sparse vegetation on dunes, 22.04.2011, leg. Huang C.-W., Luan Y., and Potapov M.B.; 3 paratypes: at the same place; 2 paratypes: China, Hainan, western coast, nearby Changhua town and ca 5 km NE from the town, in both locations by flotation of sand with plant roots in dunes of sea coast, leg. Bu Y., Huang C.-W., Kuznetsova N.A., Potapov M.B. 06.04.2011. Material is deposited in Institute of Plant Physiology & Ecology, Shanghai (holotype and two paratypes) andin Moscow State Pedagogical University (three paratypes).

#### Description.

Size about 0.4 mm. White, without eyes. Cuticle smooth. PAO about 1.7 as long as inner edge of U.III and somewhat shorter (0.8-0.9) than width of Ant.I. Outer (Fig. 23) and inner mouthparts principally as in *Pauropygus caussaneli*. Ventral side of head with 5+5 postlabial chaetae. Ant.I with about 18 chaetae, 1 ventro-basal (bms) microchaeta (dorsal bms not differentiated), and 2 thick ventral sensilla (s), short and long. Ant.II with 3 bms and 1 thick laterodistal s. Ant.III without bms and with 5 distal s of which two inner thicker and longer than outer ones. Male antennal “spurs” present.

**Figures 21–24.**
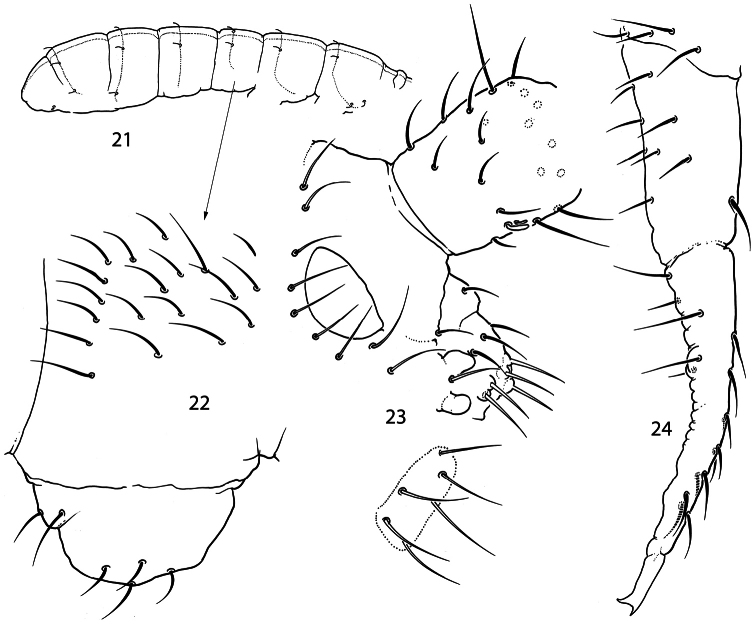
*Pauropygus pacificus* sp. n. **21** sensillar chaetotaxy **22** lateral part of Abd.I **23** anterior part of head, lateral view (group of basolateral chaetae marked, outer maxillary lobe and labial palp not shown) **24** furca, lateral view.

Thorax without ventral chaetae. Dorsal macrochaetae smooth, weakly differentiated, as 3,3,3,4 on Abd.I-IV. Medial macrochaetae on Abd.V about 0.4 as long as dens. All sensilla of thorax and medial sensilla of abdomen nearly as long as ordinary chaetae and hardly visible, lateral sensilla on Abd.III-V shorter than ordinary chaetae. Microsensilla absent. Sensillar formula 31/11221 (s), 00/000 (ms) (Fig. 21). Sensilla in posterior position. Microsensilla on Abd.I absent (Fig. 22).

Unguis of normal shape, without inner tooth, two broad unequal lateral teeth. Upper subcoxa of Leg I-III with 1,1,4 chaetae, lower subcoxa with 1,6-7,7-8. Ti.I–III with 20, 21, >25 chaetae. Distal tibiotarsal tenent chaetae on Ti.I–III (1-2-2) well developed, not clavate, about 1.1-1.2 as long as U.III. Each tibiotarsus with one additional tenent chaeta at middle part. Ti.III with one stick-like chaetae (A7) in distal ring. Tenent hairs (1,2,2) slightly longer than U.III. Pretarsus with two chaetae. Ventral tube with 4+4 laterodistal and 4 posterior chaetae in one transversal row. Tenaculum with 4+4 teeth and 1 chaeta. Anterior furcal subcoxa with 12-14 chaetae, posterior with 6 ones (rarely 5). Manubrium principally as in previous species. Dens slender, anteriorly with 10-12 chaetae. Posterior side of dens slightly crenulated in the medial part, with 5 chaetae of which 3 basal and 2 at the medial part (short inner and long outer) set together on low papillum. Mucro slender with two teeth of unequal size (Fig. 24). Ratio of manubrium: dens: mucro = 3.7–3.8: 4.0–5.0: 1. Anal lobes without microchaetae.

#### Affinity.

The complete absence of microsensilla on body of *Pauropygus pacificus* sp. n. is the second reported case among Isotomidae (so far only known in *Proisotoma minima*). This peculiarity remains the only difference between *Pauropygus pacificus* sp. n. and *Pauropygus caussaneli*. Taking into consideration also the different distribution ranges of these species we prefer to consider them as two independent species.

#### Name derivation.

The species is probably distributed in the sands all over the Pacific coast of China.

#### Distribution.

Known from two distant localities on Pacific coast of China.

##### Taxonomical remarks on genera similar to *Pauropygus*

Examination of numerous species of the *Cryptopygus* complex revealed that six genera of this complex share the posterior position of sensilla on the three first abdominal segments. Like in other taxa of Isotomidae, as for instance in *Proisotoma* complex (Potapov et al., 2006) and in species groups of *Folsomia* (Grow & Christiansen, 1976; Deharveng, 1979), the position of medial sensilla on abdominal tergites (within or well in front of posterior row of chaetae) is taxonomically very relevant at supraspecific level. The posterior position of sensilla is shared by several genera of the *Cryptopygus* complex and not necessarily indicates close relationships, but rather might be a result of convergent evolution. It is for instance the case in the genus *Micrisotoma* Bellinger, 1952 which also belongs to *Cryptopygus* complex and shows relationships with *Hemisotoma* Bagnall, 1949 in which sensilla are in mid-tergal position (type species *Isotomina thermophila*).

To figure out the relation between these genera, we propose below a key to the genera of the *Cryptopygus* complex with posterior position of sensilla on three first abdominal segments. *Pauropygus projectus* sp. n. lost posterior sensilla and can be identified by pleural fold; the loss of mentioned sensilla is probably shared with several species of *Arlea* but descriptions do not give information on these characters. The position of the odd genus *Appendisotoma* is not fully decided: six species studied by us (*Appendisotoma abiskoensis* (Agrell), *Appendisotoma bisetosa* Martynova (types), *Appendisotoma sibirica* Stebaeva (types), and three species from Far East Russia and North America) have 11 apical chaetae on tibiotarsi while other four (*Appendisotoma stebayevae* (Grinbergs), *Appendisotoma montana*(Martynova), *Appendisotoma juliannae* (Traser et al.), and one species from Kazakhstan) show common set for the subfamily (7 chaetae). We include the genus *Appendisotoma* in the key since it formally matches the diagnosis of *Cryptopygus* complex.

##### Key to genera of *Cryptopygus* complex with sensilla in posterior position on body tergites

**Table:** 

1	At least 6+6 ommatidia on head. Sensilla on abdomen subequal, Abd.I - III and Abd.IV often with more than 3 and 4 sensilla on each side, respectively (Figs 25–26). Usually dark coloured	*Appendisotoma* Stach, 1947
–	Usually blind, at most 2+2 ommatidia on head. Sensilla at the end of abdomen differentiated, Abd.I - III with no more than 2 sensilla on each side (Figs 27–29). White	2
2	Mucro falciform	*Arlea* Womersley, 1939
–	Mucro bidentate	3
3	PAO complex, with lobes. Well-marked cylindrical foil-chaetae at the end of abdomen. Anterior medial sensilla on Abd.V present (Fig. 27)	*Micrisotoma* Bellinger, 1952
–	PAO simple. Without foil-chaetae at the end of abdomen. Anterior medial sensilla on Abd.V absent (Figs 8, 28, 29)	4
4	Pleural fold on mouth cone with two finger-like processes (Fig. 20)	*Pauropygus* gen. n.
–	Pleural fold without such processes	5
5	Abd.IV with 3 sensilla on each side. Dorsal part of Abd.V with a pair of leaf-like sensilla (Fig. 28)	*Proisotomodes* Bagnall, 1949
–	Abd.IV with 2 sensilla on each side. Dorsal part of Abd.V with two pairs of long and slender sensilla (Fig. 29)	*Isotominella* Delamare Deboutteville, 1948

The key is based on examination of three species of *Pauropygus*, four of *Proisotomodes* (one undescribed), two of *Isotominella* (one undescribed), eight of *Appendisotoma* (two undescribed), and one of *Micrisotoma*.

**Figures 25–29.**
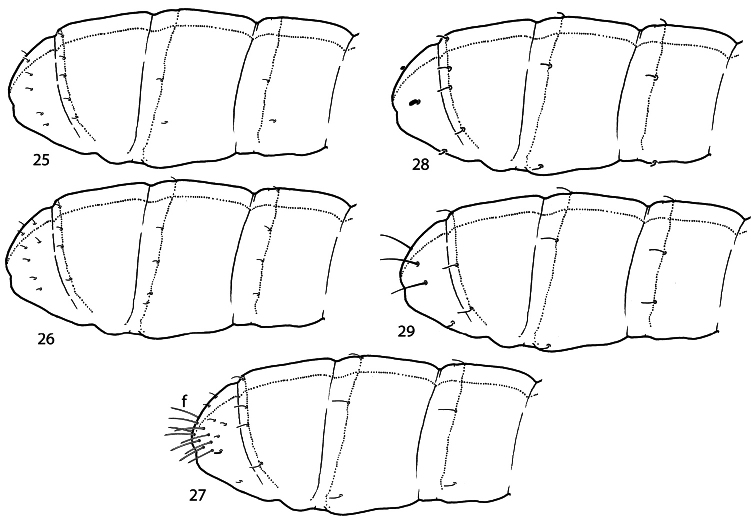
Sensillar chaetotaxy of genera of *Cryptopygus* complex with abdominal sensilla in posterior position. **25** *Appendisotoma bisetosa* Martynova, 1969 **26** *Appendisotoma abiskoensis* (Agrell, 1939) **27** *Micrisotoma achromata* Bellinger, 1952 **28** *Proisotomodes bipunctatus* (Axelson, 1903) **29** *Isotominella geophila* Delamare Deboutteville, 1948. f foil chaetae.

## Supplementary Material

XML Treatment for Pauropygus

XML Treatment for Pauropygus projectus

XML Treatment for Pauropygus caussaneli

XML Treatment for Pauropygus pacificus
